# Microsporidia, a Highly Adaptive Organism and Its Host Expansion to Humans

**DOI:** 10.3389/fcimb.2022.924007

**Published:** 2022-06-16

**Authors:** Nirin Seatamanoch, Switt Kongdachalert, Sakone Sunantaraporn, Padet Siriyasatien, Narisa Brownell

**Affiliations:** ^1^ Department of Parasitology, Faculty of Medicine, Chulalongkorn University, Bangkok, Thailand; ^2^ Vector Biology and Vector Borne Disease Research Unit, Department of Parasitology, Faculty of Medicine, Chulalongkorn University, Bangkok, Thailand

**Keywords:** microsporidia, *Trachipleistophora hominis*, zoonosis, opportunistic infection, disseminated microsporidiosis

## Abstract

Emerging infectious disease has become the center of attention since the outbreak of COVID-19. For the coronavirus, bats are suspected to be the origin of the pandemic. Consequently, the spotlight has fallen on zoonotic diseases, and the focus now expands to organisms other than viruses. Microsporidia is a single-cell organism that can infect a wide range of hosts such as insects, mammals, and humans. Its pathogenicity differs among species, and host immunological status plays an important role in infectivity and disease severity. Disseminated disease from microsporidiosis can be fatal, especially among patients with a defective immune system. Recently, there were two *Trachipleistophora hominis*, a microsporidia species which can survive in insects, case reports in Thailand, one patient had disseminated microsporidiosis. This review gathered data of disseminated microsporidiosis and *T. hominis* infections in humans covering the biological and clinical aspects. There was a total of 22 cases of disseminated microsporidiosis reports worldwide. Ten microsporidia species were identified. Maximum likelihood tree results showed some possible correlations with zoonotic transmissions. For *T.* hominis, there are currently eight case reports in humans, seven of which had Human Immunodeficiency Virus (HIV) infection. It is observed that risks are higher for the immunocompromised to acquire such infections, however, future studies should look into the entire life cycle, to identify the route of transmission and establish preventive measures, especially among the high-risk groups.

## Introduction

Microsporidiosis is an emerging infectious disease caused by a eukaryote from phylum Microsporidia. Microsporidians are capable of infecting both humans and animals depending on the species (Didier and Weiss, 2006). For human beings, the most affected population is the immunocompromised (Han and Weiss, 2017). Regarded as a highly successful parasite, microsporidia adaptability to co-live with their host is impressive. Compelling evidence demonstrated that they have undergone massive genome reduction which has resulted in the loss of considerable protein expressions and crucial organelles such as mitochondria (Wadi and Reinke, 2020). The deprivation of the components essential for survival in general living things was compensated by their remarkable ability to exploit and steal energy and food from their hosts (Barandun et al., 2019). *T. hominis* is a member of Microsporidia phylum that originated from insects. Currently, few case reports of *T. hominis* infection in humans have been reported worldwide. The most common organ involvement of *T. hominis* infection is the muscle, manifesting as myositis (Curry et al., 2005; Sundaram et al., 2019). A case report of *T. hominis* myositis has been newly reported in southern Thailand (Buppajarntham et al., 2021). A rarer form of the clinical findings is the disseminated disease, with only two cases in the literature until 2021 when a case of disseminated microsporidiosis caused by *T. hominis* has been recently reported in the South of Thailand (Ledford et al., 1985; Field et al., 1996; Siripaitoon et al., 2021). The patient was immunocompromised suffering from HIV. Therefore, we would like to conduct a review of disseminated microsporidiosis and *T. hominis* infection in human beings. The findings will provide a deeper insight into microsporidiosis specifically *T. hominis* infection encompassing clinical and molecular aspects of the disease.

## Biology and Evolution

Microsporidia is an obligate intracellular eukaryote. It can persist in the environment within the resistant chitin-shelled spore. The distinguishing characteristics that segregate them as another unique taxon include polar tube, posterior vacuole, polaroplast, and diploid chromosomes (in some species). The spore size is around 2-4 µm. The protist reproduces by sporogony in the host cell (Mahmud et al., 2017).

Its life cycle consists of merogony and sporogony stages. For merogony, the mature spore germinates the polar tube and uses it as a weapon to enter the host cell during the infective phase. The apparatus pierces through the cell membrane, paving the way for sporoplasm to enter and later on divides (Weiss et al., 2014). A study in *Encephalitozoon hellem* illustrates that the polar tube will become ejected in a stimulating environment. Polar tube protein 1 (PTP1) on the apparatus binds with the mannose binding proteins (MBP) on the host’s cell surface leading to the formation of a synapse for the entering sporoplasm. Additionally, the microsporidia’s polar tube protein 4 (PTP4) interacts with transferrin receptor 1 (TfR1) on the host’s cell which facilitates the endocytosis for the creation of the final product, parasitophorous vacuole (Han et al., 2017). The following phase is merogony, as sporoplasm proliferates within the vacuole into meronts producing multinucleate plasmodium forms. Eventually, the sporogony phase ensues, the membrane of each meront thickens and then develops into sporonts. The sporonts later divide into sporoblasts and transform into mature spores in the wait for host cell explosion to exit and infect other organisms (Vávra and Lukeš, 2013; Han and Weiss, 2017).

Microsporidia were firstly divided into three types according to their morphology, namely the Metchnikovellidae, the Chytridiopsidae, Hesseidsae, and Burkeidae, and the higher Microsporidia. The higher Microsporidia differs from one of the earliest diverging metchnikovellids in that it contains 32 conserved protein families (Mikhailov et al., 2017). However, due to certain overlapping features among groups, reallocation to a different genus for a species is not uncommon (Franzen et al., 2006; Han and Weiss, 2017). The advent of molecular analysis launched a new way to categorize these organisms and facilitated the determination of their similarities and differences among one another and from other organisms. To date, there are roughly 1400 species under nearly 200 genera of microsporidia (Vávra and Lukeš, 2013).

*T. hominis* was formerly classified in genus *Pleistophora*. Through the identification of the difference in ultrastructure, it was given a new name. *T. hominis* is distinctive from *Pleistophora* in that it lacks sporogonial plasmodium and has characteristic sporophorous vesicle walls (Hollister et al., 1996). Hollister et al. described *T. hominis* spores measuring at 4.0 x 2.4 µm with a prominent posterior vacuole. The meronts contain few nuclei while the mature spores contain 9-12 coils of polar tube (Chupp et al., 1993; Hollister et al., 1996; Weiss and Vossbrinck, 1998).

Phylogenetic analysis reveals that rozellids (cryptomycotans) are the microsporidia’s closest relative neighboring fungi (Bass et al., 2018). Apart from the correlated genetic links, a number of cryptomycotans possess certain enzymes that allow them to survive without their functioning mitochondria. This suggests that microsporidia might have emerged from these fungi (Timofeev et al., 2020).

The study of small subunit ribosomal RNA (SSU rRNA) of microsporidia revealed a great variation of genome size, ranging from 2.3 to 51.3 million base pairs (Mb) (Heinz et al., 2012; Wadi and Reinke, 2020). The phenomenon was assumed to have stemmed from the parasite’s heavy reliance upon its host resulting in the disposal of less necessary genes. The smallest microsporidia ever identified was *Encephalitozoon intestinalis* (2.3 Mb) (Corradi et al., 2010). Galindo et al. described a progressive reduction in DNA repair gene in metchnikovellids, a group of microsporidia, supporting a high evolutionary rate (Galindo et al., 2018). Microsporidia are left with substantially fewer functioning proteins subsequent to the extraordinary genome reduction. Reorientation of the ribosomal protein use was observed to make up for such genetic compaction. Furthermore, the lack of crucial energy producing-organelle like mitochondria, first discovered in *Trachipleistophora hominis*, is secondary to such genome loss as well (Williams et al., 2002; Barandun et al., 2019; Timofeev et al., 2020). Evidence demonstrated that microsporidia stole adenosine triphosphate (ATP) from its host by mitochondrial binding during residing adaptation (Hacker et al., 2014; Galindo et al., 2018). Despite the absence of a great number of proteins, the remains are sufficient for core basic metabolic pathways such as pentose phosphate and glycolysis pathways (Timofeev et al., 2020).

Unlike most microsporidia, *T. homini*s genome is relatively large consisting of 8.5 Mb. The preservation of their genes facilitated the study of the microsporidia gene reduction process. The detection of a mitochondrial protein (mitochondrial Hsp70) in a membrane bound organelle in *T. hominis* elucidated its reluctance to lose membrane organelles, confirming the effort for parasitic adjustments (Williams et al., 2002). Additionally, Ferguson et al. has recently found cell-coating in *T. hominis* that assists the microsporidia in glucose utilization at *T. hominis* and host cell interface (Ferguson and Lucocq, 2019).

The reduced genome size of the microsporidia does not directly correlate with the level of evolution as a large number of genes comes from the non-coded repetitive regions known as transposal elements (TEs) (Parisot et al., 2014). RNA inference genes always coexist with TEs, and it is suggested that RNA inference machinery is responsible for the TE activity prevention (Peyretaillade et al., 2015). RNAi machinery has been suggested to have roles in the eukaryotic defense against foreign nucleic acid such as virus, exogenous DNA, transposon, as well as gene integrity preservation. It is also claimed responsibility for drug tolerance and resistance. Due to this observation, it is inferred that expression of TEs could pose threats to the genome integrity in microsporidia in general (Heinz et al., 2012). RNAi machinery is expressed during *T. hominis* infection but is not regarded as the top 5% overall gene expression (Watson et al., 2015). Similarly, the lack of RNAi was observed in highly developed fungi. It was suspected that these organisms possessed a superior mechanism of protection to compensate for such loss (Lax et al., 2020). Further study of the presence TEs might shed light on the chronological evolution of microsporidia.

## Host Specificity

Microsporidia have a diverse host range. The first group of microsporidia was identified 160 years ago in silkworms namely *Nosema bombycis.* The microsporidia caused Pébrine or pepper disease in silkworms which, at the time, was a great threat to the silk industry (Esvaran et al., 2018). Additionally, microsporidia can infect a rather restricted host range. *Nematocida* species can only infect nematodes of *Caenorhabditis* spp. and *Oscheius tipulae.* Of note, in the same host, tissue tropism was observed among few microsporidia as well; some species infect only intestinal cells while another species infects only epithelial cells (Zhang et al., 2016). One of the exceptions is *Enterocytozoon bieneusi*, which can reside in several mammals and avians, inferring having a broad host range. Another factor attributed to the host range expansion might be the immune status. *Anncaliia algerae* was originally regarded as a parasite of mosquitoes. However, inoculation of *Anncaliia algerae* spores in athymic mice could cause infection in their connective tissue (Trammer et al., 1997). Emerging case reports of *Anncaliia algerae* in humans also proved that *Annaliia algearae* host is not limited to mosquitoes (Coyle et al., 2004; Sutrave et al., 2018). Noticeably, all the identified human cases were immunocompromised, therefore, it is assumed that there is a correlation between the poor immunological status and the higher susceptibility. In the efforts to determine the host range, the study of *E. bieneusi* specific ribosomal internal transcribed spacer (ITS) and multilocus sequence typing (MLST) proved achievable (Li et al., 2019a; Li et al., 2019b). Nonetheless, continuing efforts to further unravel the mechanism behind microsporidia host range specification should be made, especially in the concerns of public health issues (Li et al., 2019b). Furthermore, *in vitro* studies demonstrated that some microsporidia could grow in different tissues in a range of temperatures. This might be the explanation of organ tropisms such as cornea (Visvesvara, 2002).

**Figure 1:**
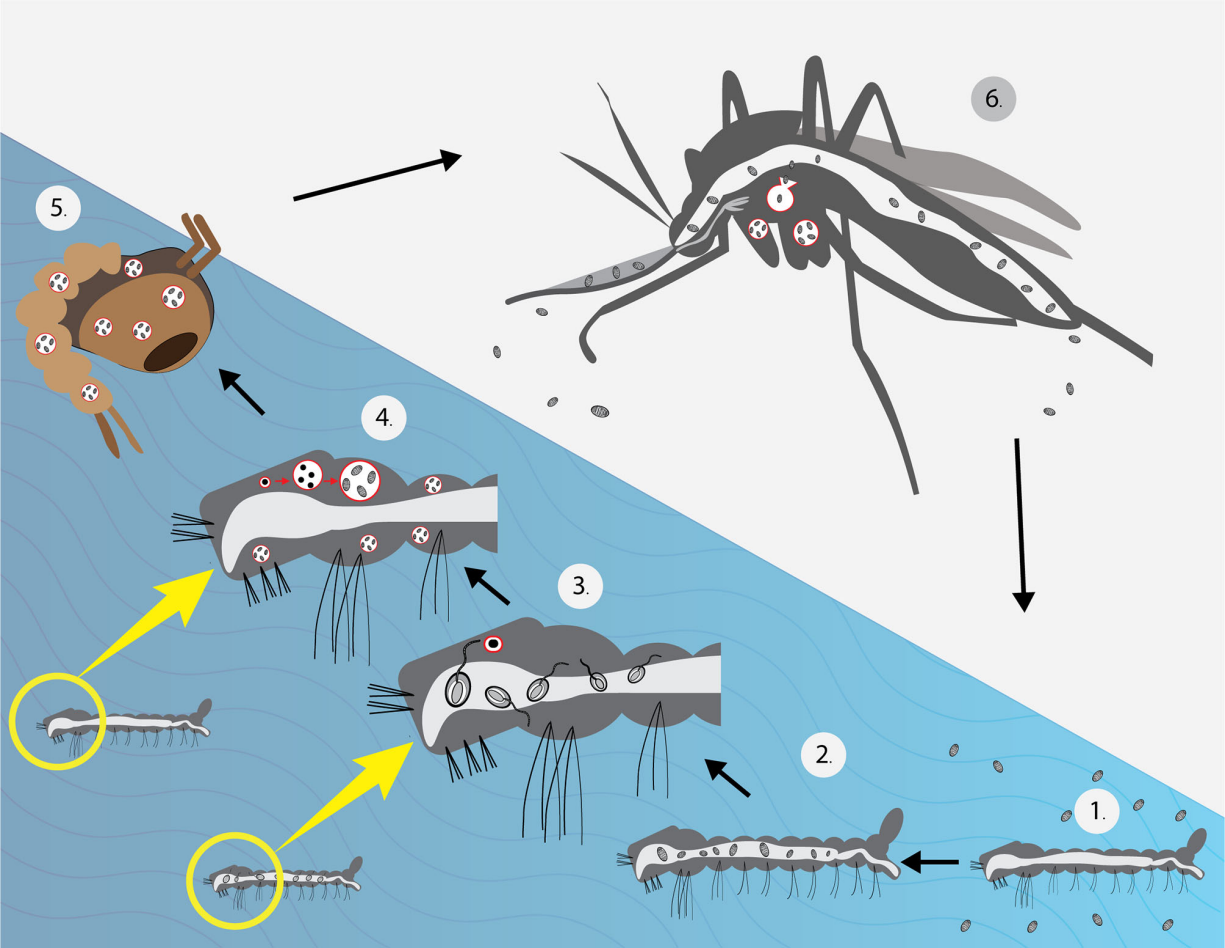
Life cycle of *T. hominis* under laboratory conditions (Weidner et al., 1999). (1) Larvae were placed in water with *T. hominis* spores. (2) Spores observed in the gut (3). After several hours, the majority of spores germinated (4). After 1 week, the larvae matured into 3^rd^ instars and showed significant infection in their muscles. (Merogony and sporogony occurred during this stage) (5). Almost all microsporidia reached the spore stage before developing into pupae. (6) Spores were found in muscles, hemocoel, proboscis and, the cecal region. A low number of spores was detected in sugar water during feeding. The mosquito image here was taken from freepik.com.

Previous phylogenetic analysis shows that *T. hominis* is close to *Vavraia culicis. V. culicis* is a parasite of anopheline and culicine mosquitoes. Despite their morphological differences, it was proved that *T. hominis* could infect mosquitoes under laboratory conditions. The protist is suspected to be transmitted by hematophagous insects similar to *Anncaliia algerae* of mosquitoes, which had reports of causing corneal infection and myositis in human beings (Cheney et al., 2000; Sutrave et al., 2018). *T. hominis* can infect larvae of both *Anopheles quadrimaculatus* and *Culex quinquefasciatus* in a laboratory setting. Similar to the infection in human beings, insect microsporidia can bear toxic effects on their hosts: reducing longevity and fertility (Becnel and Andreadis, 2014). However, the mentioned research showed that more than half of the infected mosquito larvae survived and matured into adults. The microsporidia were detected in the muscles, hemocoel, and feeding tubes. The postulated *T. hominis* life cycle in a mosquito is shown in **Figure 1**. Spores taken from the infected mosquitoes caused significant myositis in athymic mice. Furthermore, spores were passively transferred during sugar water meals, even though the number of spores was limited (Weidner et al., 1999; Mathis et al., 2005). This may imply that the mosquitoes may possess specific qualities that allow the parasites to live within their bodies with insignificant deleterious effect. Additionally, Bayesian analysis conducted in 2015 also suggested that *T. hominis* originated as a parasite of insects (Watson et al., 2015).

Other insects of the suspect for *T. hominis* ancestor hosts are the ants and bees. As mentioned, *T. hominis* genome is comparatively larger among other small microsporidia. *E. cuniculi* genome consists of merely 2.9 Mb whereas *T. hominis* genome is almost three times larger (8.5 Mb) (Katinka et al., 2001). As previously described, the factor that contributes to such a phenomenon is the density of the genome. *T. hominis* genome arrangement is substantially less dense, containing much more intergenic spacers, transposons within. Contrastingly, small-sized microsporidia such as *E. hellem*, *E. cuniliculi*, and *E. intestinalis* genes are extremely compact and bear little to no space for TEs (Peyretaillade et al., 2015). There are numerous types of TEs, such as non-LTR elements, Helitron, and piggyBac. PiggyBac jumping gene is present in ants and bees. It is also found in *T. hominis* and *Nosema ceranae* (Watson et al., 2015)*. N. ceranae* is an obligate microsporidia species in honeybees, which disrupts the physiology of the bee gut (Burnham, 2019). From genetic analysis, the piggyBac element presented in *T. hominis* was dispersed, illustrating the tendency to have been acquired independently rather than vertically. From the phylogenetic analysis, it was assumed that it obtained the genes *via* lateral gene transfer (LGT) from hymenopteran insects (Watson et al., 2015). Fish is another suspected reservoir host of *T. hominis* because it is the host for the closely related *Pleistophora ronneafiei* (Cheney et al., 2000). However, overall, there is still no solid evidence of *T. hominis* natural host.

## Microsporidia and Humans

The earliest confirmed microsporidiosis case in a human was caused by the microsporidia of insect, *Anncaliia connori* (Margileth et al., 1973; Sprague, 1974; Franzen et al., 2006; Vávra and Lukeš, 2013). Compiled evidence regarding the transmission of the microsporidia among the insects entails both horizontal and vertical means. It has been concluded that horizontal transmission can occur by 1. Cannibalistic feeding of the infected host by another host 2. Oral ingestion of contaminated materials (by dissolved dead bodies, feces, or saliva excretion of the infected in the environment), and 3. Skin exposure to the contaminated ovipositor. Two mechanisms are responsible for vertical transmission: transovarial and *via* the egg surface. Additionally, the mode of transmission differs among species (Becnel and Andreadis, 2014). As for humans, the disease supposedly spreads through fecal-oral, oral-oral routes, and inhalation of infective spores (Li et al., 2019a). Therefore, contact with reservoir hosts, such as mammals, may pose a risk for infection in a susceptible individual. Waterbody close to animal reservoirs can be the source of the resistant spores, which can remain viable for up to one year (Field and Milner, 2015). Microsporidiosis outbreak was reported in Sweden and the identified source was the sandwiches with cheese and pre-washed fresh cucumbers. It was assumed that the water used to wash the cucumbers was contaminated (Decraene et al., 2012). The direct infection from mosquitoes remains unproven as a human who had been blood-fed by a heavily infected *Anncaliia algerae* mosquito did not acquire the disease. The current assumption for the route of infection from the mosquitoes is also the consumption of contaminated water as the mosquitoes are aquatic insects (Undeen and Alger, 1976; Sutrave et al., 2018). Another hypothesis suggests that crushing the infected mosquitoes on the bite site allows disease acquisition (Coyle et al., 2004).

Almost 20 species of microsporidia have been known to cause infection in humans (Han and Weiss, 2017; Li et al., 2019b; Han et al., 2021). The most common pathogenic species is *E. bieneusi* accounting for as many as 90% of all cases (Stentiford et al., 2016; Qiu et al., 2019). It was also responsible for the previously mentioned epidemic in Sweden. Apart from the usual route, an interesting report by Smith et al. described three cases of organ recipients from a brain-dead patient with unrecognized microsporidia infection. The donor had intracranial hemorrhage resulting from complications of cranial aneurysms and arteriovenous malformations repair. All recipients suffered from a spectrum of neurological symptoms. *Encephalitozoon cuniculi* was identified *via* autopsy in one of the three recipients, other transplant centers were notified of the potential donor-derived infection in which prompt diagnosis and treatment were given to the surviving patients (Smith et al., 2017).

**Figure 2:**
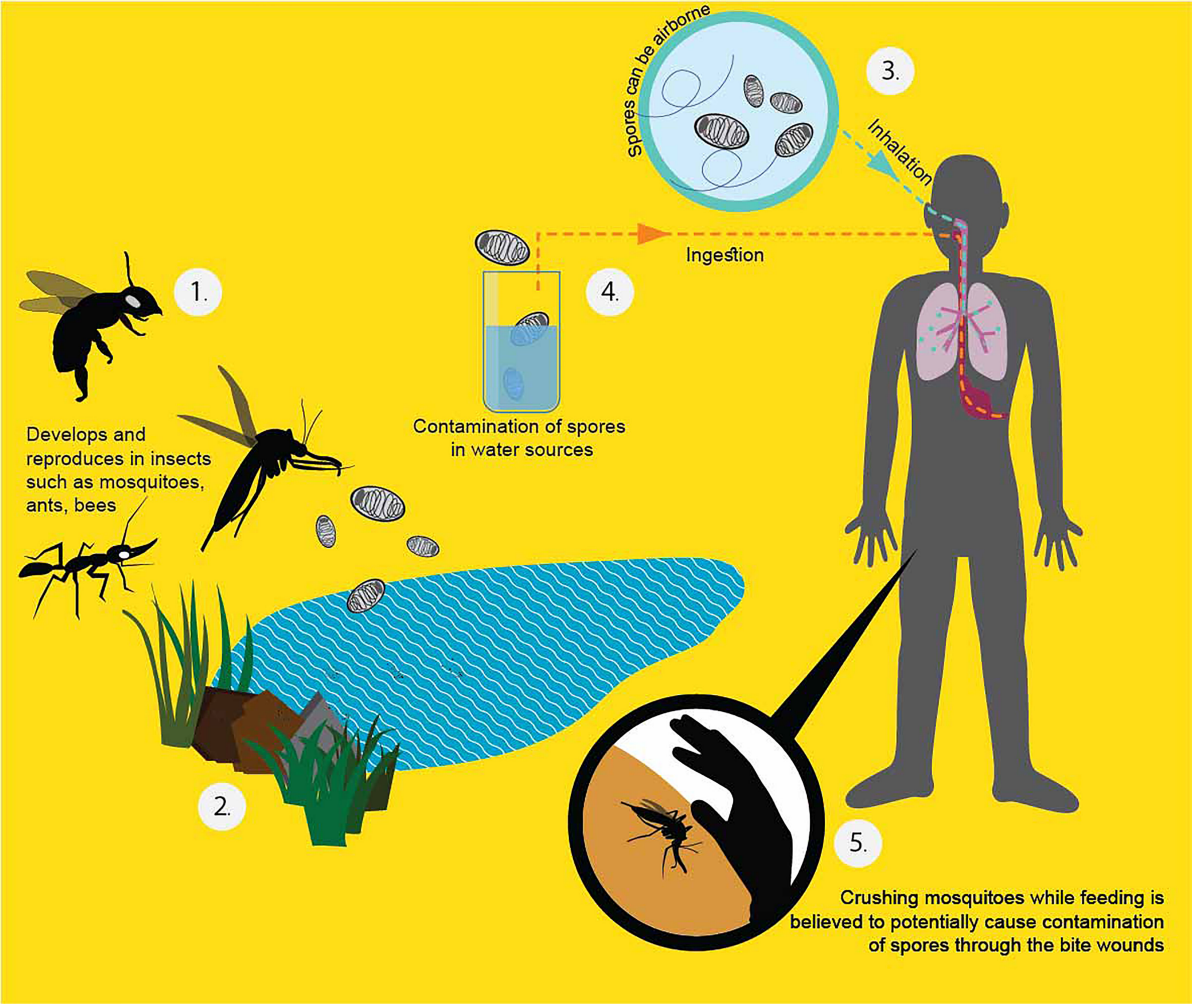
Proposed *T. hominis* transmission in human beings (Heinz et al., 2012; Becnel and Andreadis, 2014; Watson et al., 2015). From the literature, the infected potential hosts can pass the spores with their secretion or feces. The dead bodies, once decay, can also release the resistant spores to the surroundings (1). The spores can persist outside for a long time because of the protective chitin shell (2). When a healthy host ingests the spores from the environment, they become infected. People can get the spores by inhalation (3), ingestion of contaminated water (4), and directly crushing the affected insect into the bite wound (5).

*T. hominis* fails to grow in human cells at 37°C in the laboratory (Cheney et al., 2000). This supports that it might have not been well-adapted to live in human beings, which is congruent with the low number of cases in human beings until present. Therefore, it may be inferred that immune deficient patients acquired the parasite after becoming immunologically depressed, and that the infection was not the result of hidden infection. From the gathered information, it is assumed that the insects are the source of infection. From its genetic relatedness with *V. culicis* and hymenopterans, a proposed diagram of *T. hominis* transmission to humans is shown in **Figure 2**.  

## Zoonosis Presumption From Phylogenetic Analysis

**Figure 3:**
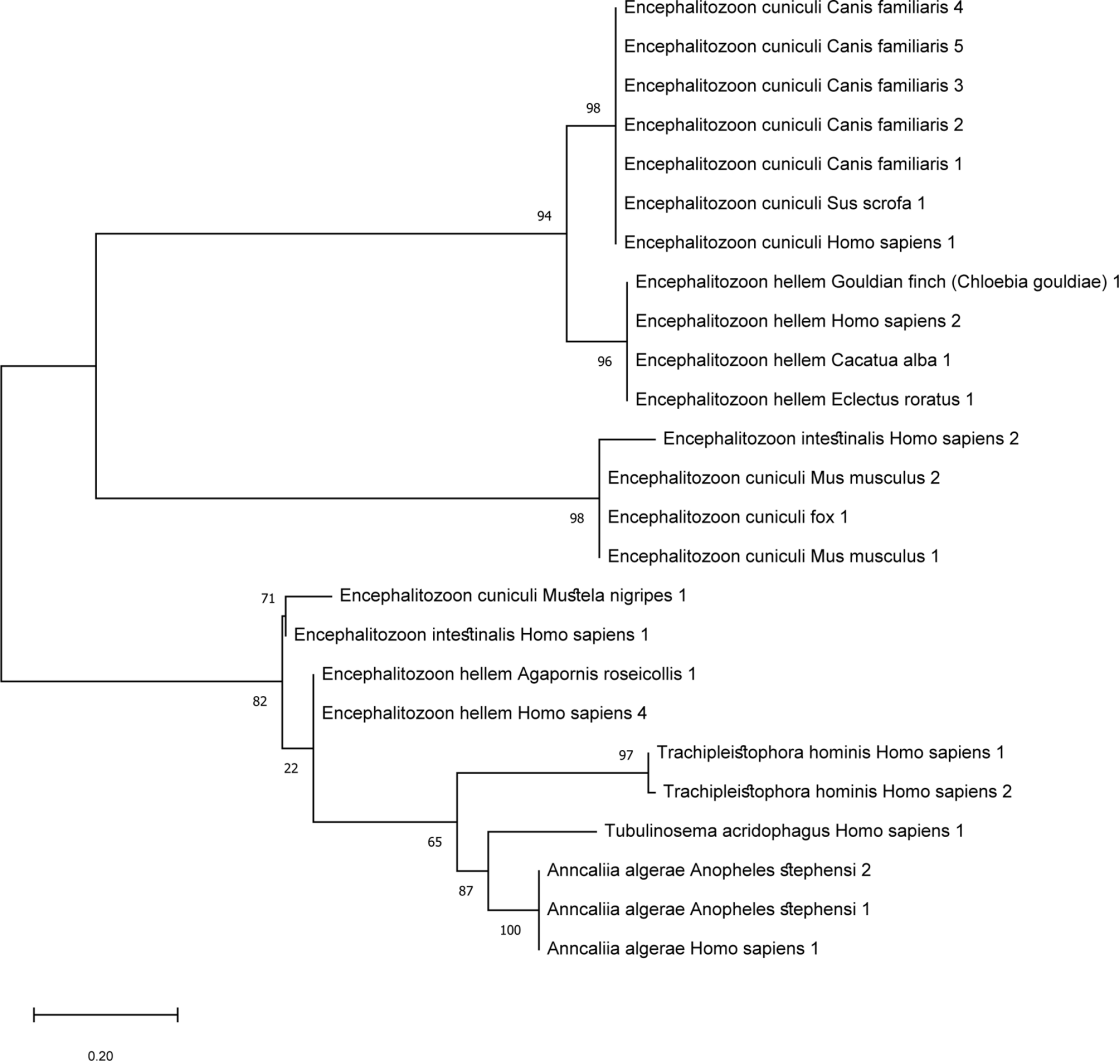
Maximum likelihood tree of microsporidia that cause disseminated disease in humans and sequences of the same species of other hosts were included. ML tree was constructed in Mega software version X using Kimura-2 parameter model. The sequences included were based on microsporidia species with reports of causing disseminated diseases in humans. Other nucleotide sequences detected in other organisms were included to find links of zoonosis. *T. hominis* species are closely related to *A. algerae* which is congruent with previous reports.

The tree analysis of microsporidia which reported to have caused disseminated disease in human beings is displayed in **Figure 3**. The sequences of the identified microsporidia in other hosts were also included to find clues of zoonotic transmission. For *E. cuniculi*, all the hosts are mammals such as dogs (*Canis familiaris*), and wild boars (*Sus scrofa*). The sequences that are in proximity to *E. cuniculi* obtained from humans are of dogs. *E. cuniculi* infection is prevalent among pets such as dogs, cats, and rabbits, and is considered zoonotic. Genetic analysis of an infected patient in Switzerland revealed an identical genotype of the prevalent species among rabbits in the area (Weber et al., 1997). For *E. hellem*, the majority of the hosts are of bird species and reported cases in humans are adjacent to the sequences from avian, consistent with previous reports (Polonais et al., 2010). *T. hominis* aligns closely to *A. algerae*, which has reports of infection in mosquitoes (*Anopheles stephensi*) and humans. *T. hominis* position illustrated here correlated with the assumption that the protist might have originated from insects.

## Looming Zoonosis Rise

From our review, it might be indicated that mosquitoes, ants, and bees may serve as a vector for microsporidia of different species. Given that these arthropods might have the capacity to transmit microsporidia to humans, other zoonoses carried by these arthropods may also deserve attention. Geographics heavily influence the prevalence of specific vector-borne diseases as climate greatly affects the vector’s population. For tropical regions, the dominating vectors include mosquitoes and sand flies, which are responsible for, for example, malaria and leishmaniasis respectively (Carvalho et al., 2017). *Plasmodium knowlesi* is a common pathogen for simian malarias in humans. Various *Anopheles* species that are regarded as vectors of *P. knowlesi* are reported in a myriad of countries in Southeast Asia. The ability to infect sympatric species may be a factor that has made the infection more widespread (van de Straat et al., 2022). Such a phenomenon might also occur in some particular anopheles-borne microsporidia whereby it could be carried by multiple species that dwell in the same geographical area. **Table 1** illustrated the host spectrum of some microsporidia. It is noticeable that a few could be detected in both insects and humans. Apart from cross-species vectors, the case of cross-genus vectors is observed for *Leishmania* spp. *Leishmania* parasites were recently reported in *Culicoides* biting midges apart from the widely known *Phlebotomine* sand flies in various countries, as well as Thailand (Dougall et al., 2011; Sunantaraporn et al., 2021). The cross-genus finding has highlighted the parasite’s adaptability and/or the host’s competence. The circulations of pathogens among animals and humans might be more extensive than previously known. Several factors may be held accountable. The ongoing human invasion of the forests can significantly lead to increased exposure to disease reservoirs and vectors. The patient of the disseminated *T. hominis* case from the South of Thailand was a rubber tree cultivation worker (Siripaitoon et al., 2021). Working in such an environment may have facilitated parasite acquisition. Additionally, it is believed that human activities that result in climate change have greatly contributed to the rise of emerging infectious diseases in various regions around the world (Morand and Lajaunie, 2021; Rupasinghe et al., 2022). To elucidate further, the amount of rainfall can play a crucial part in some insects’ abundance, for instance (Kishimoto-Yamada and Itioka, 2015; Siripaitoon et al., 2021). The risks may be higher due to the possibility of contamination of resistant spores in the environment for microsporidia. Due to deforestation, excessive migration, and climate change, the expansion of these vector-borne diseases is imminent. Contributing factors should be promptly identified to help slow down the process.

**Table 1:** Host spectrum of *Microsporidia* spp. which can cause systemic infection and other major clinical findings.

Microsporidia species	Genomic size (Mb)	Host	Major clinical features in humans	Reference
*E. cuniculi*	2.28	Human, avian, non-human primate, rodent, carnivore	Cerebral infection, disseminated disease, diarrhea	(Weber et al., 1997; Mathis et al., 2005)
*E. hellem*	2.25	Human, avian, non-human primate, rodent, carnivore	Disseminated disease, keratoconjunctivitis	(Schwartz et al., 1992; Scaglia et al., 1994; Mathis et al., 2005)
*E. intestinalis*	2.21	Human, avian, non-human primate, rodent, carnivore	Disseminated disease, diarrhea	(Mathis et al., 2005; Flores et al., 2021)
*T. hominis*	8.49	Human, insect	Myositis	(Weidner et al., 1999; Buppajarntham et al., 2021; Siripaitoon et al., 2021)
*A. algerae*	13.82	Human, insect	Myositis, keratitis	(Visvesvara et al., 1999; Coyle et al., 2004; Sutrave et al., 2018)
*T. acridophagus*	N/A	Human, insect	Myositis, skin nodules	(Choudhary et al., 2011; Meissner et al., 2012; Connors et al., 2017)

N/A, non-applicable.

## Clinical Picture Including Disseminated Disease in Humans

Microsporidia usually cause asymptomatic or self-resolving infection in healthy individuals (Didier and Weiss, 2006; Han and Weiss, 2017). Sak et al. observed high rates of positive antibodies against microsporidia in asymptomatic persons. In addition, spores were detected in either urine or stool samples in 100% of the participants over the research period. Therefore, it was assumed that the prevalence of microsporidia might be higher than previously estimated in the normal population. Co-infection of multiple microsporidia species was found in a few individuals as well (Sak et al., 2011). By contrast, the clinical manifestations in an immunocompromised host can be markedly severe. It is still of debate whether the disease is a sequela of a hidden infection or newly acquired (Teachey et al., 2004). HIV patients whose CD4+ T cell level is below 100 cells/mm^3^ are predisposed to the infection (CDC et al.). A meta-analysis in 2018 by Qiu et al. estimated an overall prevalence of *E. bieneusi* in China at 8.1% among AIDS patients (Qiu et al., 2019). Microsporidiosis was also reported in transplant patients who were on immunosuppressants as well as patients who were on chemotherapy (Mahmood et al., 2003; Carlson et al., 2004). Mice models demonstrated immunological reactions against microsporidia infection, which highlighted the importance of innate and adaptive responses. Both responses were claimed crucial for parasite prevention and elimination (Tomazatos et al., 2020). Nonetheless, we are seeing emerging evidence of infections among immunocompetent hosts as well (Didier and Weiss, 2011).

A common clinical manifestation in an immune defect host is persistent diarrhea. The parasites enter *via* the oral route and proliferate in the epithelial intestinal cells, reducing the digestive surface area, which results in diarrhea (Didier and Weiss, 2006). Other forms of infection include keratitis, sinusitis, encephalitis, and myositis. Some major clinical findings are displayed in **Table 1**. However, the infection can manifest as a disseminated form attacking multiple organs (Didier, 2005). Regardless of the onset of immune deficiency, systemic microsporidiosis affects people of all ages. The first case of disseminated microsporidiosis was confirmed in a four-month-old infant with thymic aplasia (Margileth et al., 1973). From our case review of 22 disseminated microsporidiosis, we found seven cases were of HIV patients, 11 cases were of post-transplant hosts, and one was of an immunocompetent host (**Table 2**). Suankratay et al. reported a disseminated microsporidiosis case in a 43-year-old male patient with no underlying diseases. The causative agent was *Endoreticulatus* spp. (Suankratay et al., 2012). From our gathered data, the most commonly affected systems in disseminated cases were the urinary system and respiratory system accounting for 64% (15/22 cases) and 59% (14/22 cases), respectively. Other organ involvement included the gastrointestinal system, muscle, central nervous system, skin, eyes, bone marrow, sinus, thyroid, parathyroid glands, and adrenal glands. Furthermore, the mortality rate stands at roughly 50%.

**Table 2:** Disseminated microsporidiosis cases.

No.	Author		Year	Infectious Agent	Age/Sex	Outcome	Treatment		Clinical Manifestation		Host		CD4+ (cells/m^3^)	Country	Organ involvement
1	(Margileth et al. (1973)		1973	*A. connori*	4 mo/ M	Death	Antibiotic		Diarrhea, vomiting, irritability, lethargy, fever, maculopapular rash with pustules			Hypogamma- globulinemia	N/A	U.S.A.	Myocardium, liver, diaphragm, adrenals, GI tract
2	Schwartz et al. (1992)		1992	*E. hellem*	30/M	Death	N/A		Prostatitis, hematuria, flank pain			AIDS	32	U.S.A.	KUB tract, RS tract, conjunctivae
3	Scaglia et al. (1994)		1994	*E. hellem*	32/M	N/A	N/A		N/A			AIDS	N/A	Italy	Kidneys, lungs, liver
4	Yachnis et al. (1996)		1996	*T. anthropophthera*	8/F	Death	Phenobarbital		Seizures, aphasia, diminished consciousness, hallucinations, inability to walk, difficulty breathing			AIDS ***	N/A	U.S.A.	Kidneys, liver, brain, thyroid, parathyroid, heart, pancreas
					Sex				Manifestation					
5	Field et al. (1996)	1996		*T. hominis*	34/M	Recovered but died of HIV	Albendazole sulfadiazine pyrimethamine		Myalgias, diplopia, lethargy, weight loss, cough, fever odynophagia		AIDS		N/A		Australia	Skeletal muscle, conjunctivae, nasal sinuses
6	Gamboa-Dominguez et al. (2003)	2001		*E. cuniculi*	42/M	Recovered but relapse albendazole reinstituted after one year	Albendazole		Fever, productive cough, thoracic pain, weakness, hematuria, diarrhea		Post-KT (terminal GN and hypertension)		N/A	Mexico	Kidneys, GI tract, liver, skin,
7	Mohindra et al. (2002)	2002		*E. cuniculi*	45/F	Death	Albendazole Fumagillin eye drops		Bilateral keratoconjunctivitis, allograft tenderness, fever, generalized seizure		Post-KT (Chronic glomerulone-phritis)		N/A	Canada	Kidney, lungs, GI tract, conjunctivae, brain	
8	Svedhem et al. (2002) (Case 1)	2002		*E. intestinalis*	60/M	Death	Albendazole		Nausea, weight loss, diarrhea		AIDS		200	Sweden		Lungs, GI tract	
9	Svedhem et al. (2002) (Case 2)	2002		*E. intestinalis*	39/M	Died from EBV encephalopathy and B-cell lymphoma	Albendazole		Conjunctivitis, rhinitis, sinusitis fever, diarrhea, cough		AIDS		N/A	Sweden		Lungs, GI tract, conjunctivae, nose, sinuses	
No.	Author	Year		Infectious Agent	Age/ Sex	Outcome	Treatment		Clinical Manifestation		Host		CD4+ (cells/m^3^)	Country		Organ involvement	
10	Carlson et al. (2004)	2004		*Encephalitozoon* spp.	43/M	Death	N/A		Fever, graft tenderness, anuria, diarrhea		Post-pancreas, kidney transplant (T1DM with ESRD)		N/A	U.S.A.	Kidneys, liver, GI tract, heart, brain, diaphragm	
11	Talabani et al. (2010)	2009		*E. cuniculi*	38/F	Recovery	Albendazole		Fever, cough, abdominal pain, anorexia		Post-KT (ESRD due to IgA nephropathy)		N/A		France	Kidneys, lungs
12	George et al. (2012)	2012		*Encephalitozoon* spp.	57/M	Recovery	Albendazole		Pneumonia		Post-KT (ESRD due to DN)		N/A		Australia	Kidneys, lungs	
13	Suankratay et al. (2012)	2012		*Endoreticulatus* spp.	43/F	Recovered but died of aspiration pneumonia	Albendazole		Difficulty swallowing, weight loss, leg swelling		Immunocompetent		N/A		Thailand	Kidneys, muscle, bone marrow
No.	Author		Year	Infectious Agent	Age/ Sex	Outcome	Treatment	Clinical Manifestation		Host		CD4+ (cells/m^3^)		Country		Organ involvement
14	Meissner et al. (2012)		2012	*T. acridophagus*	33/F	Death	N/A	Respiratory failure, jaundice, diarrhea, ascites		Post-HSCT (MM)		N/A		U.S.A.	Liver, lungs, skin
15	Nagpal et al. (2013)		2013	*E. cuniculi*	68/F	Recovery	Albendazole	Nonproductive cough, fever, chills		Post-KT due to ESRD (ADPKD)		N/A		U.S.A.		Kidneys, lungs
16	Boileau et al. (2016)		2016	*A. algerae*	49/M	Recovery	Albendazole pyrimethamine TMP-SMX Fumagilin	Fever, limb pain		CLL on ibrutinib		190		Canada	Deltoids, triceps, quadriceps, brain
17	Smith et al. (2017) (Case 1)		2017	*E. cuniculi*	N/A (M)	Recovery	Albendazole	Bilateral tremor, light-headedness, blurry vision, headache		Post-liver transplant (HCV cirrhosis and HCC)		N/A		U.S.A.		Kidneys, brain
					Sex						Manifseation				
18	Smith et al. (2017) (Case 2)		2017	*E. cuniculi*	N/A (F)	Recovery	Albendazole	Fever, fatigue, pain in wrists and shins, elbows		Post kidney and heart transplant**		N/A		U.S.A.	Kidneys, brain
19	Smith et al. (2017) (Case 3)		2017	*E. cuniculi*	N/A (M)	Death	Antimicrobial drugs	Generalized weakness, confusion, fever		Post-KT*		N/A		U.S.A.		Kidneys, brain
20	Connors et al. (2017)		2017	*T. acridophagus*	58/F	Recovered but expired due to CLL	Albendazole	Widespread skin nodules, nonproductive cough		Undergoing chemotherapy conditioning for HSCT		N/A		Canada	Skin, liver, lungs
21	Anderson et al. (2019)		2019	*A. algerae*	60/M	Death	Albendazole itraconazole clindamycin	Papular rash on lower extremities		Post-pancreas allograft transplant (T1DM)		N/A		U.S.A.	Kidneys, lungs, finger, tongue, lower extremity
22	Siripaitoon et al. (2021)		2021	*T. hominis*	29/F	Death	Albendazole TPM/SMX clindamycin	Incapacitating myalgias, fever, lethargy		AIDS		15		Thailand	Muscle, bone marrow

M, Male, G, Female, GI, gastrointestinal, KUB, Kidney, ureter, and bladder, T1DM, Type 1 Diabetes Mellitus, T2DM, Type 2 Diabetes Mellitus, DN, Diabetic nephropathy, GN, glomerulonephritis, HCC, hepatocellular carcinoma, CLL, Chronic Lymphocytic Leukemia, TMP-SMX, Trimethoprim/sulfamethoxazole, ESRD, End stage renal disease, HSCT, hematopoietic stem cell transplantation, ADPKD, Autosomal dominant polycystic kidney disease, MM, Multiple Myeloma, HCV, Hepatitis C Virus, KT, Kidney Transplant, N/A, non-applicable; *ESRD due to T2DM **coronary vasculopathy and calcineurin inhibitor–induced nephropathy *** HIV (congenitally acquired), hemangiopericytoma (metastatic to diaphragm and intestine) *E. cuniculi*: *Encephalitozoon cuniculi*, *A. algerae*: *Anncaliia algerae*, *T. acridophagus*: *Tubulinosema acridophagus*, *Encephalitozoon* spp.: *Encephalitozoon* species, *E. intestinalis*: *Encephalitozoon intestinalis*, *T. hominis*: *Trachipleistophora hominis*, *E. hellem*: *Encephalitozoon hellem*, *Endoreticulatus* spp.: *Endoreticulatus* species, *T. anthropophthera*: *Trachipleistophora anthropophthera*, *A. connori*: *Anncaliia connori*.

The reported causative agents of disseminated microsporidiosis cases were *Anncaliia algerae, Tubulinosema acridophagus, Encephalitozoon cuniculi, Encephalitozoon hellem, Endoreticulatus* spp.*, Encephalitozoon* spp.*, Encephalitozoon intestinalis, Trachipleistophora hominis, Trachipleistophora anthropophthera, and Anncaliia connori.* The most common microsporidia species was *E. cuniculi* (7/22 cases). All cases with *E. cuniculi* had urinary system involvement. As opposed to the assumption, the most prevalent *E. bieneusi* has no record of systemic manifestations.

There have been eight case reports of *T. hominis* including two from Thailand by Siripaitoon et al. and Suankratay et al., as demonstrated in **Table 3** (Buppajarntham et al., 2021; Siripaitoon et al., 2021). The most reported clinical manifestation for *T. hominis* infection is myositis (6 out of 8 cases). The second most common presentation is respiratory symptoms (3 out of 8 cases). Four patients had disseminated disease including one with sole myositis but of distant areas (myocardium, skeletal muscles). The causative agent in reports by Chupp et al., Grau et al., and presumably Ledford et al. was modified from *Pleistophora ronneafiei* (*Pleistophora* spp.) to *T. hominis* due to subsequent ultrastructural characteristics discovery (Ledford et al., 1985; Chupp et al., 1993; Grau et al., 1996; Hollister et al., 1996). It was observed that the diagnosis establishment was challenging in some settings due to low awareness. Patient’s immune status plays an important role. Only one out of eight patients had no immune defect, and the pathogen was exclusively restricted to the eyes for the case (Rauz et al., 2004). Unlike some other microsporidia, *T. hominis* likely lacks tissue specificity. One patient with disseminated *T. hominis* also had keratitis. The infectibility in both corneal and stromal cells of *T. hominis* implies that it is not cell-specific (Field et al., 1996; Moshirfar et al., 2020). A study by Koudela et al. illustrated that *T. hominis* did not have tissue specificity in severe combined immunodeficient (SCID) mice and simple contamination on conjunctivae and subcutaneous inoculation, which mimicked insect bite, could lead to generalized infection (Koudela et al., 2004).

**Table 3:** *Trachipleistophora hominis* cases.

No.	Author	Year	Age/Sex	Outcome	Treatment	ClinicalManifestation	Host	CD4+ (cells/m^3^)	Country	Organ involvement
1	Ledford et al. (1985)	1985	20/M	Recovery	TPM/SMX	Generalized muscle weakness, fever, weight loss lymphadenopathy	AIDS	N/A	U.S.A.	Sinuses, muscle, testicular failure, hypoandrogenism
2	Chupp et al. (1993)	1993	33/M	Death	Pyrimethamine clindamycin	Fever, productive cough, myalgias, weakness	AIDS	N/A	Haiti	Skeletal muscle
3	Grau et al. (1996)	1996	N/A	Article in Spanish	Article in Spanish	Myositis, fever	AIDS	N/A	Article in Spanish	Muscle
4	Field et al. (1996)	1996	34/M	Recovered but died of progressive HIV	Albendazole sulfadiazine pyrimethamine	Myalgias, diplopia, lethargy, weight loss, odynophagia, cough, fever	AIDS	N/A	Australia	Skeletal muscle, conjunctivae, nasal sinuses
5	Rauz et al. (2004)	2004	22/M	Recurrence	Topical Fumagillin PHMB 0.02% albendazole	Photophobia, blurred vision	Immuno-competent	N/A	Ghana	Eye stroma
6	Curry et al. (2005)	2005	47/M	Death (Suicide)	N/A	Left upper lobe pneumonia	AIDS	30	Australia	Pectoral muscles, myocardium
7	Siripaitoon et al. (2021)	2021	29/F	Death	Albendazole TPM/SMX clindamycin	Incapacitating myalgias, fever, lethargy	AIDS	15	Thailand	Muscle, bone marrow
8.	Buppajarntham et al. (2021)	2021	45/M	Recovery	Albendazole pyrimethamine clindamycin	Myalgia	AIDS	12	Thailand	Skeletal muscle

N/A, non-applicable.

## Diagnosis

**Figure 4:**
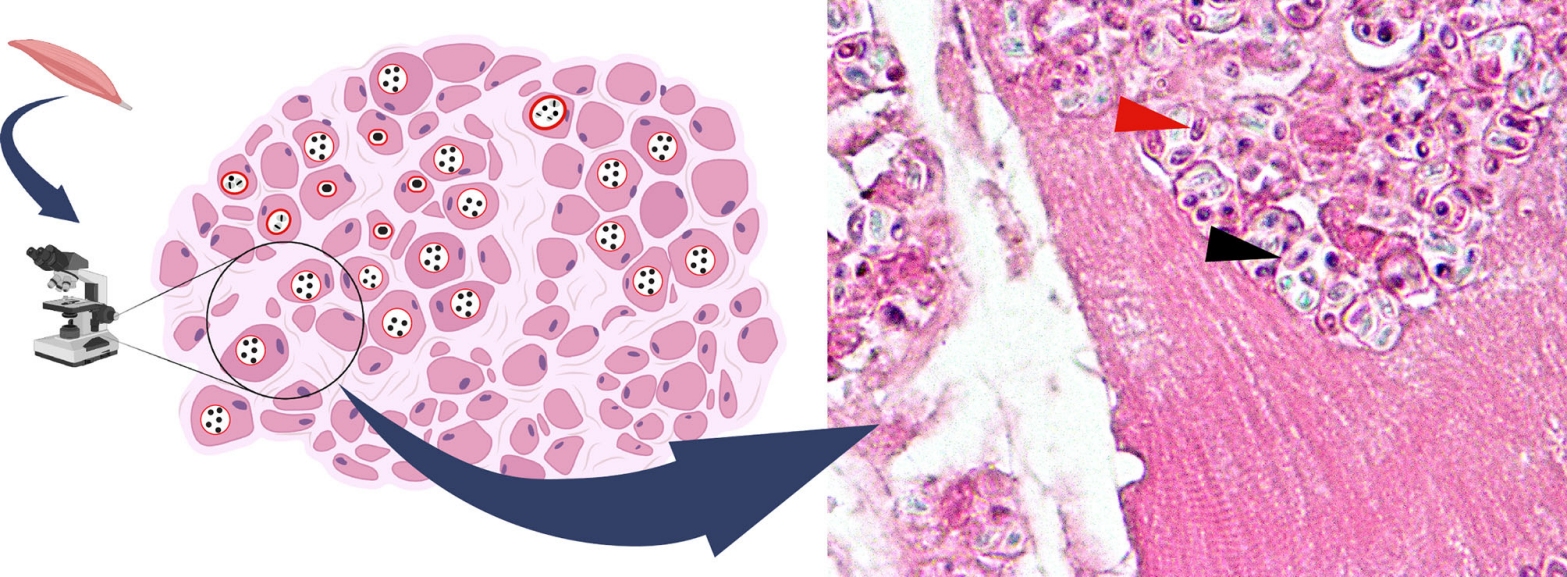
Hematoxylin & eosin stain of *T. hominis* in skeletal muscle (magnification X1000). Spores observed in circular spaces (parasitophorous vacuoles) in muscle fiber (black arrow). Spore with a characteristic belt-like stripe (red arrow). This image was created with BioRender (http://biorender.com/).

The diagnosis can be achieved by a number of staining techniques, antigen or antibody detection, transmission electron microscopy (TEM), and nucleic analysis (Valenčáková and Sučik, 2020). The gold standard diagnostic method is TEM. The technique allows the identification of the unique ultrastructural features which offers species determination. However, the technique is both laborious and time-consuming. It also requires specialized expertise for characteristic polar tube identification. Staining with modified trichrome and other conventional stains such as Warthin-starry silver is the more approachable method. The organism is thick-walled and contains a mid-line crossing belt as shown in **Figure 4**. Chemo-fluorescent stains (for example, Uvitex 2B, Calcofluor White) enhance the wall visibility but can be misinterpreted with fungi (Ghosh and Weiss, 2009). For fecal or urine sampling, multiple specimen collection over a time course may increase yields as spore excretion timing differs from one person to another (Sak et al., 2011). One precaution for the staining technique is the misidentification between microsporidia and *Toxoplasma gondii* cyst as they are similar in both appearance and size (Dubey et al., 1998). Due to indistinct clinical features in the immunocompromised of both infections and lower awareness for microsporidiosis, few patients were initially misdiagnosed with toxoplasmosis instead. Request for further specific microsporidia stains is recommended if a patient is diagnosed with toxoplasmosis (Chupp et al., 1993; Teachey et al., 2004; Buppajarntham et al., 2021). Antigen and antibody detection techniques should be used along with other techniques. Unreliability of the antibody-based detection is subject to cross reactions and difficulties in establishing the onset of infection (acute or past infection). Therefore, it is more utilized in the screening for hidden infections of organ donors. Furthermore, species identification is crucial for treatment as the drug of choice varies upon the species and organs affected (CDC et al.). Even though detailed morphology can be examined by TEM, for some close species such as *E. cuniculi* and *E. hellem*, TEM alone might be inadequate. The nucleic acid analysis comes in useful. rRNA of microsporidia contains both conserved and variable genes and thus are used as the target for PCR-based techniques (Ghosh and Weiss, 2009). PCR primers for SSU rRNA for specific species were described by Weiss et al. (Weiss and Vossbrinck, 1998). Additionally, large subunit (LSU), and ITS genes from the rRNA can be used to identify novel species of microsporidia as well. The majority of specimen types can be directly used for PCR analysis by regular DNA extraction procedure, however, stool samples need mechanical disruption prior to DNA amplification (Weiss and Vossbrinck, 1998; Ghosh and Weiss, 2009).

## Treatments

The mainstay medication for microsporidiosis is albendazole and fumagillin. Albendazole inhibits β-tubulin which halts the parasite mitosis. Fumagillin targets methionine aminopeptidase type 2 (MetAP2), which results in increased lipid incorporation in the parasite membrane and leads to cell breakage and death. The agent also plays a role in RNA synthesis blockade (Anane and Attouchi, 2010). Albendazole covers a narrower spectrum of microsporidia when compared to fumagillin. Despite fumagillin efficacy, its toxicity of bone marrow suppression undermines its attractiveness. Albendazole cannot contain *E. bieneusi* infection but fortunately, nitazoxanide was proved effective (Saffo and Mirza, 2019). New drug targets have been introduced for treatment, but to date, only meagre data is available for their effectiveness (Han and Weiss, 2018). For intestinal microsporidiosis, albendazole 400 mg orally three times daily for two weeks or twice daily for four weeks is recommended (Mahmud et al., 2017). The main challenge of the treatment of disseminated cases is the poor immunological status of the patients. It is observed in many cases that albendazole use was extended until the patients achieved significant clinical improvement and clinical specimens were free of the parasites. It is also recommended that, in HIV patients, discontinuing the medication should be considered after their CD4+ has reached 100 cells/mm^3^ while the patients are concurrently taking antiretrovirals to boost their immune system (CDC et al.; Talabani et al., 2010). Additionally, some physicians decided to give a lifelong albendazole prescription to a post kidney transplant patient (George et al., 2012). If the immunological status of the patient cannot be fully restored, the benefits of prolonged albendazole use might outweigh the risks. There is still no indication for chemical prophylaxis against microsporidia, thus maintaining good personal hygiene and consuming fully cooked food might be an optimal solution to lower the infection risks.

Apart from *E. bieneusi*, albendazole was claimed effective against other microsporidial infections, as well as *T. hominis* (CDC et al.). Treatment outcomes varied in *T. hominis* patients. Due to a limited number of cases, it is challenging to determine its efficacy. For HIV patients with *T. hominis* myositis treated with albendazole, three cases reported improvement (one recovered, but two died of progressive HIV disease). *In vitro* study of albendazole in mouse myoblast cell line revealed unpromising results. It was observed that the spore stage was unaffected and led to the parasite recovery. The assumption was that *T. hominis* β-tubulin, a target of albendazole, may differ from other microsporidia (Lafranchi-Tristem et al., 2001). Other medications used were pyrimethamine, bactrim, etc. Pyrimethamine was used in patients that firstly were diagnosed with myositis of *Toxoplasma gondii* (Chupp et al., 1993; Buppajarntham et al., 2021). The patients who received the pyrimethamine got worse or did not respond to the medication (Chupp et al., 1993; Field et al., 1996; Buppajarntham et al., 2021). The patient who received bactrim and other antimicrobials excluding albendazole reported slight improvement (Ledford et al., 1985). Early treatment before numerous spore formations might provide better results. Similar to other microsporidiosis cases, immune restoration should be of equal importance (CDC et al.).

Microsporidia is a parasite of various hosts. Evidence have shown its potential for zoonotic transmission in which patients with depressed immune are at a higher risk of acquiring the infection. *T. hominis* should be suspected when the diagnosis of toxoplasmosis is made. Molecular diagnosis may be helpful in such cases. As a highly adaptive parasite, we might see a greater number of cases of microporidiosis in human beings in the future. The role of the insects as a vector should be further explored for the rise of *T. hominis* infections in Thailand.

## Author Contributions

Conception and design: NB, PS, NS, and SS. Drafting of the manuscript: NS and SK. Drawing of figures: NS, SK, and SS. Conceiving and critical revision of the manuscript for important intellectual content: NB, PS. All authors contributed to the article and approved the submitted version.

## Conflict of Interest

The authors declare that the research was conducted in the absence of any commercial or financial relationships that could be construed as a potential conflict of interest.

## Publisher’s Note

All claims expressed in this article are solely those of the authors and do not necessarily represent those of their affiliated organizations, or those of the publisher, the editors and the reviewers. Any product that may be evaluated in this article, or claim that may be made by its manufacturer, is not guaranteed or endorsed by the publisher.

## References

[B1] Anane S. Attouchi H. (2010). Microsporidiosis: Epidemiology, Clinical Data and Therapy. Gastroenterol. Clin. Biol. 34 (8-9), 450–464. doi: 10.1016/j.gcb.2010.07.003 20702053

[B2] Anderson N. W. Muehlenbachs A. Arif S. Bruminhent J. Deziel P. J. Razonable R. R.. (2019). A Fatal Case of Disseminated Microsporidiosis Due to Anncaliia Algerae in a Renal and Pancreas Allograft Recipient. Open Forum Infect. Dis. 6 (7), ofz285. doi: 10.1093/ofid/ofz285 31304191 PMC6612885

[B3] Barandun J. Hunziker M. Vossbrinck C. R. Klinge S. (2019). Evolutionary Compaction and Adaptation Visualized by the Structure of the Dormant Microsporidian Ribosome. Nat. Microbiol. 4 (11), 1798–1804. doi: 10.1038/s41564-019-0514-6 31332387 PMC6814508

[B4] Bass D. Czech L. Williams B. A. P. Berney C. Dunthorn M. Mahé F.. (2018). Clarifying the Relationships Between Microsporidia and Cryptomycota. J. Eukaryot. Microbiol. 65 (6), 773–782. doi: 10.1111/jeu.12519 29603494 PMC6282948

[B5] Becnel J. J. Andreadis T. G. (2014). “Microsporidia in Insects,” in Microsporidia. (USA: John Wiley & Sons, Inc.) 521–570.

[B6] Boileau M. Ferreira J. Ahmad I. Lavallée C. Qvarnstrom Y. Dufresne S. F. (2016). Successful Treatment of Disseminated Anncaliia Algerae Microsporidial Infection With Combination Fumagillin and Albendazole. Open Forum Infect. Dis. 3 (3), ofw158. doi: 10.1093/ofid/ofw158 27704013 PMC5047403

[B7] Buppajarntham A. Atikankul T. Putaporntip C. Jongwutiwes S. Suankratay C. (2021). Myositis Caused by Trachipleistophora Hominis in a Person With Human Immunodeficiency Virus: The First Case in Thailand. Open Forum Infect. Dis. 8 (12), ofab494. doi: 10.1093/ofid/ofab494 34877363 PMC8643666

[B8] Burnham A. J. (2019). Scientific Advances in Controlling Nosema Ceranae (Microsporidia) Infections in Honey Bees (Apis Mellifera). Front. Vet. Sci. 6 (79), 79. doi: 10.3389/fvets.2019.00079 30931319 PMC6428737

[B9] Carlson J. R. Li L. Helton C. L. Munn R. J. Wasson K. Perez R. V.. (2004). Disseminated Microsporidiosis in a Pancreas/Kidney Transplant Recipient. Arch. Pathol. Lab. Med. 128 (3), e41–e43. doi: 10.5858/2004-128-e41-DMIAKT 14987135

[B10] Carvalho B. M. Rangel E. F. Vale M. M. (2017). Evaluation of the Impacts of Climate Change on Disease Vectors Through Ecological Niche Modelling. Bull. Entomol. Res. 107 (4), 419–430. doi: 10.1017/s0007485316001097 27974065

[B11] CDC NIH IDSA Panel on Opportunistic Infections in Adults and Adolescents with HIV Guidelines for the Prevention and Treatment of Opportunistic Infections in Adults and Adolescents With HIV: Recommendations From the Centers for Disease Control and Prevention, the National Institutes of Health, and the HIV Medicine Association of the Infectious Diseases Society of America (Accessed 7 May 2021).

[B12] Cheney S. A. Lafranchi-Tristem N. J. Canning E. U. (2000). Phylogenetic Relationships of Pleistophora-Like Microsporidia Based on Small Subunit Ribosomal DNA Sequences and Implications for the Source of Trachipleistophora Hominis Infections. J. Eukaryot. Microbiol. 47 (3), 280–287. doi: 10.1111/j.1550-7408.2000.tb00048.x 10847345

[B13] Choudhary M. M. Metcalfe M. G. Arrambide K. Bern C. Visvesvara G. S. Pieniazek N. J.. (2011). Tubulinosema Sp. Microsporidian Myositis in Immunosuppressed Patient. Emerging Infect. Dis. 17 (9), 1727–1730. doi: 10.3201/eid1709.101926 PMC3322067 21888805

[B14] Chupp G. L. Alroy J. Adelman L. S. Breen J. C. Skolnik P. R. (1993). Myositis Due to Pleistophora (Microsporidia) in a Patient With AIDS. Clin. Infect. Dis. 16 (1), 15–21. doi: 10.1093/clinids/16.1.15 8448294

[B15] Connors W. J. Carson J. A. Chan W. W. Parkins M. D. (2017). Albendazole-Responsive Disseminated Tubulinosema Acridophagus in a Patient With Chronic Lymphocytic Leukaemia. Clin. Microbiol. Infect. 23 (9), 684–685. doi: 10.1016/j.cmi.2017.04.002 28400339

[B16] Corradi N. Pombert J. F. Farinelli L. Didier E. S. Keeling P. J. (2010). The Complete Sequence of the Smallest Known Nuclear Genome From the Microsporidian Encephalitozoon Intestinalis. Nat. Commun. 1, 77. doi: 10.1038/ncomms1082 20865802 PMC4355639

[B17] Coyle C. M. Weiss L. M. Rhodes L. V. Cali A. Takvorian P. M. Brown D. F.. (2004). Fatal Myositis Due to the Microsporidian Brachiola Algerae, a Mosquito Pathogen. New Engl. J. Med. 351 (1), 42–47. doi: 10.1056/nejmoa032655 15229306 PMC3109631

[B18] Curry A. Beeching N. J. Gilbert J. D. Scott G. Rowland P. L. Currie B. J. (2005). Trachipleistophora Hominis Infection in the Myocardium and Skeletal Muscle of a Patient With AIDS. J. Infect. 51 (3), e139–e144. doi: 10.1016/j.jinf.2004.11.006 16230193

[B19] Decraene V. Lebbad M. Botero-Kleiven S. Gustavsson A. M. Löfdahl M. (2012). First Reported Foodborne Outbreak Associated With Microsporidia, Sweden, October 2009. Epidemiol. Infect. 140 (3), 519–527. doi: 10.1017/s095026881100077x 21733266 PMC3267097

[B20] Didier E. S. (2005). Microsporidiosis: An Emerging and Opportunistic Infection in Humans and Animals. Acta Trop. 94 (1), 61–76. doi: 10.1016/j.actatropica.2005.01.010 15777637

[B21] Didier E. S. Weiss L. M. (2006). Microsporidiosis: Current Status. Curr. Opin. Infect. Dis. 19 (5), 485–492. doi: 10.1097/01.qco.0000244055.46382.23 16940873 PMC3109650

[B22] Didier E. S. Weiss L. M. (2011). Microsporidiosis: Not Just in AIDS Patients. Curr. Opin. Infect. Dis. 24 (5), 490–495. doi: 10.1097/QCO.0b013e32834aa152 21844802 PMC3416021

[B23] Dougall A. M. Alexander B. Holt D. C. Harris T. Sultan A. H. Bates P. A.. (2011). Evidence Incriminating Midges (Diptera: Ceratopogonidae) as Potential Vectors of Leishmania in Australia. Int. J. Parasitol. 41 (5), 571–579. doi: 10.1016/j.ijpara.2010.12.008 21251914

[B24] Dubey J. P. Lindsay D. S. Speer C. A. (1998). Structures of Toxoplasma Gondii Tachyzoites, Bradyzoites, and Sporozoites and Biology and Development of Tissue Cysts. Clin. Microbiol. Rev. 11 (2), 267–299. doi: 10.1128/CMR.11.2.267 9564564 PMC106833

[B25] Esvaran V. G. Gupta T. Nayaka A. R. N. Sivaprasad V. Ponnuvel K. M. (2018). Molecular Characterization of Nosema Bombycis Methionine Aminopeptidase 2 (MetAP2) Gene and Evaluation of Anti-Microsporidian Activity of Fumagilin-B in Silkworm Bombyx Mori. 3 Biotech. 8 (9), 386–386. doi: 10.1007/s13205-018-1411-z PMC6108961 30175023

[B26] Ferguson S. Lucocq J. (2019). The Invasive Cell Coat at the Microsporidian Trachipleistophora Hominis–Host Cell Interface Contains Secreted Hexokinases. Microbiologyopen 8, 4. doi: 10.1002/mbo3.696 PMC6460350 30051624

[B27] Field A. S. Marriott D. J. Milliken S. T. Brew B. J. Canning E. U. Kench J. G.. (1996). Myositis Associated With a Newly Described Microsporidian, Trachipleistophora Hominis, in a Patient With AIDS. J. Clin. Microbiol. 34 (11), 2803–2811. doi: 10.1128/JCM.34.11.2803-2811.1996 8897186 PMC229407

[B28] Field A. S. Milner D. A. Jr (2015). Intestinal Microsporidiosis. Clin. Lab. Med. 35 (2), 445–459. doi: 10.1016/j.cll.2015.02.011 26004651

[B29] Flores J. Takvorian P. M. Weiss L. M. Cali A. Gao N. (2021). Human Microsporidian Pathogen Encephalitozoon Intestinalis Impinges on Enterocyte Membrane Trafficking and Signaling. J. Cell Sci. 134 (5), jcs253757. doi: 10.1242/jcs.253757 33589497 PMC7938802

[B30] Franzen C. Nassonova E. S. Scholmerich J. Issi I. V. (2006). Transfer of the Members of the Genus Brachiola (Microsporidia) to the Genus Anncaliia Based on Ultrastructural and Molecular Data. J. Eukaryot. Microbiol. 53, 1, 26–35. doi: 10.1111/j.1550-7408.2005.00066.x 16441582

[B31] Galindo L. J. Torruella G. Moreira D. Timpano H. Paskerova G. Smirnov A.. (2018). Evolutionary Genomics of Metchnikovella Incurvata (Metchnikovellidae): An Early Branching Microsporidium. Genome Biol. Evol. 10 (10), 2736–2748. doi: 10.1093/gbe/evy205 30239727 PMC6190962

[B32] Gamboa-Dominguez A. De Anda J. Donis J. Ruiz-Maza F. Visvesvara G. S. Diliz H. (2003). Disseminated Encephalitozoon Cuniculi Infection in a Mexican Kidney Transplant Recipient. Transplantation 75 (11), 1898–1900. doi: 10.1097/01.Tp.0000064623.57821.22 12811252

[B33] George B. Coates T. McDonald S. Russ G. Cherian S. Nolan J.. (2012). Disseminated Microsporidiosis With Encephalitozoon Species in a Renal Transplant Recipient. Nephrol. (Carlton) 17 Suppl 1, 5–8. doi: 10.1111/j.1440-1797.2012.01580.x 22497646

[B34] Ghosh K. Weiss L. M. (2009). Molecular Diagnostic Tests for Microsporidia. Interdiscip. Perspect. Infect. Dis. 2009, 926521. doi: 10.1155/2009/926521 19657457 PMC2719812

[B35] Grau A. Valls M. E. Williams J. E. Ellis D. S. Muntané M. J. Nadal C. (1996). [Myositis Caused by Pleistophora in a Patient With AIDS]. Med. Clin. (Barc) 107 (20), 779–781. doi: 10.1093/clinids/16.1.15 9019606

[B36] Hacker C. Howell M. Bhella D. Lucocq J. (2014). Strategies for Maximizing ATP Supply in the Microsporidian Encephalitozoon Cuniculi: Direct Binding of Mitochondria to the Parasitophorous Vacuole and Clustering of the Mitochondrial Porin VDAC. Cell Microbiol. 16 (4), 565–579. doi: 10.1111/cmi.12240 24245785 PMC4233961

[B37] Han B. Pan G. Weiss L. M. (2021). Microsporidiosis in Humans. Clin. Microbiol. Rev. 34, 4, e00010–e00020. doi: 10.1128/CMR.00010-20 PMC8404701 34190570

[B38] Han B. Polonais V. Sugi T. Yakubu R. Takvorian P. M. Cali A.. (2017). The Role of Microsporidian Polar Tube Protein 4 (PTP4) in Host Cell Infection. PloS Pathog. 13 (4), e1006341. doi: 10.1371/journal.ppat.1006341 28426751 PMC5413088

[B39] Han B. Weiss L. M. (2017). Microsporidia: Obligate Intracellular Pathogens Within the Fungal Kingdom. Microbiol. Spectr. 5 (2). doi: 10.1128/microbiolspec.FUNK-0018-2016 PMC5613672 28944750

[B40] Han B. Weiss L. M. (2018). Therapeutic Targets for the Treatment of Microsporidiosis in Humans. Expert Opin. Ther. Targets 22 (11), 903–915. doi: 10.1080/14728222.2018.1538360 30336698 PMC6300147

[B41] Heinz E. Williams T. A. Nakjang S. Noël C. J. Swan D. C. Goldberg A. V.. (2012). The Genome of the Obligate Intracellular Parasite Trachipleistophora Hominis: New Insights Into Microsporidian Genome Dynamics and Reductive Evolution. PloS Pathog. 8 (10), e1002979. doi: 10.1371/journal.ppat.1002979 23133373 PMC3486916

[B42] Hollister W. S. Canning E. U. Weidner E. Field A. S. Kench J. Marriott D. J. (1996). Development and Ultrastructure of Trachipleistophora Hominis N.G., N.Sp. After *In Vitro* Isolation From an AIDS Patient and Inoculation Into Athymic Mice. Parasitology 112 (Pt 1), 143–154. doi: 10.1017/s0031182000065185 8587798

[B43] Katinka M. D. Duprat S. Cornillot E. Méténier G. Thomarat F. Prensier G.. (2001). Genome Sequence and Gene Compaction of the Eukaryote Parasite Encephalitozoon Cuniculi. Nature 414 (6862), 450–453. doi: 10.1038/35106579 11719806

[B44] Kishimoto-Yamada K. Itioka T. (2015). How Much Have We Learned About Seasonality in Tropical Insect Abundance Since Wolda, (1988)? Entomol. Sci. 18, 4, 407–419. doi: 10.1111/ens.12134

[B45] Koudela B. Vávra J. Canning E. U. (2004). Experimental Infection of Severe Combined Immunodeficient (SCID) Mice With the Human Microsporidian Trachipleistophora Hominis. Parasitology 128 (Pt 4), 377–384. doi: 10.1017/s0031182003004645 15151142

[B46] Lafranchi-Tristem N. J. Curry A. Cheney S. A. Canning E. U. (2001). Growth of Trachipleistophora Hominis (Microsporidia: Pleistophoridae) in C2,C12 Mouse Myoblast Cells and Response to Treatment With Albendazole. Folia Parasitol. (Praha) 48 (3), 192–200. doi: 10.14411/fp.2001.032 11699654

[B47] Lax C. Tahiri G. Patiño-Medina J. A. Cánovas-Márquez J. T. Pérez-Ruiz J. A. Osorio-Concepción M.. (2020). The Evolutionary Significance of RNAi in the Fungal Kingdom. Int. J. Mol. Sci. 21 (24), 9348. doi: 10.3390/ijms21249348 PMC7763443 33302447

[B48] Ledford D. K. Overman M. D. Gonzalvo A. Cali A. Mester S. W. Lockey R. F. (1985). Microsporidiosis Myositis in a Patient With the Acquired Immunodeficiency Syndrome. Ann. Intern. Med. 102 (5), 628–630. doi: 10.7326/0003-4819-102-5-628 3920941

[B49] Li W. Feng Y. Santin M. (2019a). Host Specificity of Enterocytozoon Bieneusi and Public Health Implications. Trends Parasitol. 35 (6), 436–451. doi: 10.1016/j.pt.2019.04.004 31076351

[B50] Li W. Feng Y. Zhang L. Xiao L. (2019b). Potential Impacts of Host Specificity on Zoonotic or Interspecies Transmission of Enterocytozoon Bieneusi. Infect. Genet. Evol. 75, 104033. doi: 10.1016/j.meegid.2019.104033 31494271

[B51] Mahmood M. N. Keohane M. E. Burd E. M. (2003). Pathologic Quiz Case: A 45-Year-Old Renal Transplant Recipient With Persistent Fever. Arch. Pathol. Lab. Med. 127 (4), e224–e226. doi: 10.5858/2003-127-e224-PQCAYO 12683908

[B52] Mahmud R. Lim Y. A. L. Amir A. (2017). “"Microsporidia,",” in Medical Parasitology (Cham: Springer International Publishing), 71–74.

[B53] Margileth A. M. Strano A. J. Chandra R. Neafie R. Blum M. McCully R. M. (1973). Disseminated Nosematosis in an Immunologically Compromised Infant. Arch. Pathol. 95 (3), 145–150. 4686506

[B54] Mathis A. Weber R. Deplazes P. (2005). Zoonotic Potential of the Microsporidia. Clin. Microbiol. Rev. 18 (3), 423–445. doi: 10.1128/cmr.18.3.423-445.2005 16020683 PMC1195965

[B55] Meissner E. G. Bennett J. E. Qvarnstrom Y. da Silva A. Chu E. Y. Tsokos M.. (2012). Disseminated Microsporidiosis in an Immunosuppressed Patient. Emerging Infect. Dis. 18 (7), 1155–1158. doi: 10.3201/eid1807.120047 PMC3376806 22709509

[B56] Mikhailov K. V. Simdyanov T. G. Aleoshin V. V. (2017). Genomic Survey of a Hyperparasitic Microsporidian Amphiamblys Sp. (Metchnikovellidae). Genome Biol. Evol. 9 (3), 454–467. doi: 10.1093/gbe/evw235 27694476 PMC5381614

[B57] Mohindra A. R. Lee M. W. Visvesvara G. Moura H. Parasuraman R. Leitch G. J.. (2002). Disseminated Microsporidiosis in a Renal Transplant Recipient. Transpl. Infect. Dis. 4 (2), 102–107. doi: 10.1034/j.1399-3062.2002.01011.x 12220248

[B58] Morand S. Lajaunie C. (2021). Outbreaks of Vector-Borne and Zoonotic Diseases Are Associated With Changes in Forest Cover and Oil Palm Expansion at Global Scale Front. Vet. Sci. 8, 661063. doi: 10.3389/fvets.2021.661063 33842581 PMC8024476

[B59] Moshirfar M. Somani S. N. Shmunes K. M. Espandar L. Gokhale N. S. Ronquillo Y. C.. (2020). A Narrative Review of Microsporidial Infections of the Cornea. Ophthalmol. Ther. 9 (2), 265–278. doi: 10.1007/s40123-020-00243-z 32157613 PMC7196102

[B60] Nagpal A. Pritt B. S. Lorenz E. C. Amer H. Nasr S. H. Cornell L. D.. (2013). Disseminated Microsporidiosis in a Renal Transplant Recipient: Case Report and Review of the Literature. Transpl. Infect. Dis. 15 (5), 526–532. doi: 10.1111/tid.12119 23947513

[B61] Parisot N. Pelin A. Gasc C. Polonais V. Belkorchia A. Panek J.. (2014). Microsporidian Genomes Harbor a Diverse Array of Transposable Elements That Demonstrate an Ancestry of Horizontal Exchange With Metazoans. Genome Biol. Evol. 6 (9), 2289–2300. doi: 10.1093/gbe/evu178 25172905 PMC4202319

[B62] Peyretaillade E. Boucher D. Parisot N. Gasc C. Butler R. Pombert J. F.. (2015). Exploiting the Architecture and the Features of the Microsporidian Genomes to Investigate Diversity and Impact of These Parasites on Ecosystems. Heredity 114 (5), 441–449. doi: 10.1038/hdy.2014.78 25182222 PMC4815508

[B63] Polonais V. Mazet M. Wawrzyniak I. Texier C. Blot N. El Alaoui H.. (2010). The Human Microsporidian Encephalitozoon Hellem Synthesizes Two Spore Wall Polymorphic Proteins Useful for Epidemiological Studies. Infect. Immun. 78 (5), 2221–2230. doi: 10.1128/iai.01225-09 20231418 PMC2863520

[B64] Qiu L. Xia W. Li W. Ping J. Ding S. Liu H. (2019). The Prevalence of Microsporidia in China : A Systematic Review and Meta-Analysis. Sci. Rep. 9 (1), 3174. doi: 10.1038/s41598-019-39290-3 30816168 PMC6395699

[B65] Rauz S. Tuft S. Dart J. K. G. Bonshek R. Luthert P. Curry A. (2004). Ultrastructural Examination of Two Cases of Stromal Microsporidial Keratitis. J. Med. Microbiol. 53 (Pt 8), 775–781. doi: 10.1099/jmm.0.45524-0 15272065

[B66] Rupasinghe R. Chomel B. B. Martinez-Lopez B. (2022). Climate Change and Zoonoses: A Review of the Current Status, Knowledge Gaps, and Future Trends. Acta Trop. 226, 106225. doi: 10.1016/j.actatropica.2021.106225 34758355

[B67] Saffo Z. Mirza N. (2019). Successful Treatment of Enterocytozoon Bieneusi Gastrointestinal Infection With Nitazoxanide in a Immunocompetent Patient. IDCases 18, e00586. doi: 10.1016/j.idcr.2019.e00586 31388488 PMC6669369

[B68] Sak B. Kváč M. Kučerová Z. Květoňová D. Saková K. (2011). Latent Microsporidial Infection in Immunocompetent Individuals - a Longitudinal Study. PloS Negl. Trop. Dis. 5 (5), e1162. doi: 10.1371/journal.pntd.0001162 21629721 PMC3101169

[B69] Scaglia M. Sacchi L. Gatti S. Bernuzzi A. M. Polver Pde P. Piacentini I.. (1994). Isolation and Identification of Encephalitozoon Hellem From an Italian AIDS Patient With Disseminated Microsporidiosis. Apmis 102 (11), 817–827. doi: 10.1111/j.1699-0463.1994.tb05240.x 7833001

[B70] Schwartz D. A. Bryan R. T. Hewan-Lowe K. O. Visvesvara G. S. Weber R. Cali A. (1992). Disseminated Microsporidiosis (Encephalitozoon Hellem) and Acquired Immunodeficiency Syndrome. Autopsy Evidence for Respiratory Acquisition. Arch. Pathol. Lab. Med. 116 (6), 660–668. 1616428

[B71] Siripaitoon P. Seatamanoch N. Brownell N. Kongdachalert S. Hortiwakul T. Phumee A. (2021). Case Report: First Report of Disseminated Trachipleistophora Hominis Infection in an AIDS Patient From Thailand. Am. J. Trop. Med. Hyg. 105 (5), 1198–1201. doi: 10.4269/ajtmh.21-0585 34460423 PMC8592187

[B72] Smith R. M. Muehlenbachs A. Schaenmann J. Baxi S. Koo S. Blau D.. (2017). Three Cases of Neurologic Syndrome Caused by Donor-Derived Microsporidiosis. Emerging Infect. Dis. 23 (3), 387–395. doi: 10.3201/eid2303.161580 PMC5382757 28220747

[B73] Sprague V. (1974). Nosema Connori N. Sp., a Microsporidian Parasite of Man. Trans. Am. Microscopical. Soc. 93 (3), 400–403. doi: 10.2307/3225442 4212205

[B74] Stentiford G. D. Becnel J. Weiss L. M. Keeling P. J. Didier E. S. Williams B. P.. (2016). Microsporidia - Emergent Pathogens in the Global Food Chain. Trends Parasitol. 32 (4), 336–348. doi: 10.1016/j.pt.2015.12.004 26796229 PMC4818719

[B75] Suankratay C. Thiansukhon E. Nilaratanakul V. Putaporntip C. Jongwutiwes S. (2012). Disseminated Infection Caused by Novel Species of Microsporidium, Thailand. Emerging Infect. Dis. 18 (2), 302–304. doi: 10.3201/eid1802.111319 PMC3310463 22305387

[B76] Sunantaraporn S. Thepparat A. Phumee A. Sor-Suwan S. Boonserm R. Bellis G.. (2021). Culicoides Latreille (Diptera: Ceratopogonidae) as Potential Vectors for *Leishmania Martiniquensis* and Trypanosoma Sp. In Northern Thailand. PloS Negl. Trop. Dis. 15 (12), e0010014. doi: 10.1371/journal.pntd.0010014 34910720 PMC8673663

[B77] Sundaram T. G. Aggarwal A. Ganguly S. Iangngap E. K. Marak R. S. K. Gupta L. (2019). Microsporidial Myositis in Adult-Onset Immunodeficiency: Case-Based Review. Rheumatol. Int. 39 (11), 1995–2003. doi: 10.1007/s00296-019-04439-w 31501996

[B78] Sutrave G. Maundrell A. Keighley C. Jennings Z. Brammah S. Wang M. X.. (2018). Anncaliia Algerae Microsporidial Myositis, New South Wales, Australia. Emerg. Infect. Dis. 24 (8), 1528–1531. doi: 10.3201/eid2408.172002 30014835 PMC6056123

[B79] Svedhem V. Lebbad M. Hedkvist B. Del Aguila C. Hedman P. Larsson R.. (2002). Disseminated Infection With Encephalitozoon Intestinalis in AIDS Patients: Report of 2 Cases. Scand. J. Infect. Dis. 34 (9), 703–705. doi: 10.1080/00365540210147598 12374372

[B80] Talabani H. Sarfati C. Pillebout E. van Gool T. Derouin F. Menotti J. (2010). Disseminated Infection With a New Genovar of Encephalitozoon Cuniculi in a Renal Transplant Recipient. J. Clin. Microbiol. 48 (7), 2651–2653. doi: 10.1128/JCM.02539-09 20463169 PMC2897537

[B81] Teachey D. T. Russo P. Orenstein J. M. Didier E. S. Bowers C. Bunin N. (2004). Pulmonary Infection With Microsporidia After Allogeneic Bone Marrow Transplantation. Bone Marrow Transplant. 33 (3), 299–302. doi: 10.1038/sj.bmt.1704327 14628080

[B82] Timofeev S. Tokarev Y. Dolgikh V. (2020). Energy Metabolism and its Evolution in Microsporidia and Allied Taxa. Parasitol. Res. 119 (5), 1433–1441. doi: 10.1007/s00436-020-06657-9 32200463

[B83] Tomazatos A. Jöst H. Schulze J. Spînu M. Schmidt-Chanasit J. Cadar D.. (2020). Blood-Meal Analysis of Culicoides (Diptera: Ceratopogonidae) Reveals a Broad Host Range and New Species Records for Romania. Parasites Vectors 13 (1), 79. doi: 10.1186/s13071-020-3938-1 32066493 PMC7027113

[B84] Trammer T. Dombrowski F. Doehring M. Maier W. A. Seitz H. M. (1997). Opportunistic Properties of Nosema Algerae (Microspora), a Mosquito Parasite, in Immunocompromised Mice. J. Eukaryot. Microbiol. 44 (3), 258–262. doi: 10.1111/j.1550-7408.1997.tb05709.x 9183715

[B85] Undeen A. H. Alger N. E. (1976). Nosema Algerae: Infection of the White Mouse by a Mosquito Parasite. Exp. Parasitol. 40 (1), 86–88. doi: 10.1016/0014-4894(76)90068-0 950003

[B86] Valenčáková A. Sučik M. (2020). Alternatives in Molecular Diagnostics of Encephalitozoon and Enterocytozoon Infections. J. Fungi 6, 3, 114. doi: 10.3390/jof6030114 PMC7558530 32707956

[B87] van de Straat B. Sebayang B. Grigg M. J. Staunton K. Garjito T. A. Vythilingam I.. (2022). Zoonotic Malaria Transmission and Land Use Change in Southeast Asia: What is Known About the Vectors. Malar J. 21 (1), 109. doi: 10.1186/s12936-022-04129-2 35361218 PMC8974233

[B88] Vávra J. Lukeš J. (2013). “Chapter Four - Microsporidia and ‘The Art of Living Together,” in Advances in Parasitology. Ed. Rollinson D. (UK: Academic Press), 253–319. 10.1016/B978-0-12-407706-5.00004-6 23548087

[B89] Visvesvara G. S. (2002). *In Vitro* Cultivation of Microsporidia of Clinical Importance. Clin. Microbiol. Rev. 15 (3), 401–413. doi: 10.1128/cmr.15.3.401-413.2002 12097248 PMC118077

[B90] Visvesvara G. S. Belloso M. Moura H. Da Silva A. J. Moura I. N. Leitch G. J.. (1999). Isolation of Nosema Algerae From the Cornea of an Immunocompetent Patient. J. Eukaryot. Microbiol. 46 (5), 10s. 10519226

[B91] Wadi L. Reinke A. W. (2020). Evolution of Microsporidia: An Extremely Successful Group of Eukaryotic Intracellular Parasites. PloS Pathog. 16 (2), e1008276. doi: 10.1371/journal.ppat.1008276 32053705 PMC7017984

[B92] Watson A. K. Williams T. A. Williams B. A. Moore K. A. Hirt R. P. Embley T. M. (2015). Transcriptomic Profiling of Host-Parasite Interactions in the Microsporidian Trachipleistophora Hominis. BMC Genomics 16, 983. doi: 10.1186/s12864-015-1989-z 26589282 PMC4654818

[B93] Weber R. Deplazes P. Flepp M. Mathis A. Baumann R. Sauer B.. (1997). Cerebral Microsporidiosis Due to Encephalitozoon Cuniculi in a Patient With Human Immunodeficiency Virus Infection. N. Engl. J. Med. 336, 7, 474–478. doi: 10.1056/nejm199702133360704 9017940

[B94] Weidner E. Canning E. U. Rutledge C. R. Meek C. L. (1999). Mosquito (Diptera: Culicidae) Host Compatibility and Vector Competency for the Human Myositic Parasite Trachipleistophora Hominis (Phylum Microspora). J. Med. Entomol. 36 (4), 522–525. doi: 10.1093/jmedent/36.4.522 10467783

[B95] Weiss L. M. Delbac F. Hayman J. R. Pan G. Dang X. Zhou Z. (2014). “The Microsporidian Polar Tube and Spore Wall,” in Microsporidia. (USA: John Wiley & Sons, Inc.), 261–306.

[B96] Weiss L. M. Vossbrinck C. R. (1998). “Microsporidiosis: Molecular and Diagnostic Aspects,” in Advances in Parasitology. Ed. Tzipori S. (UK: Academic Press), 351–395. 10.1016/s0065-308x(08)60127-x 9554079

[B97] Williams B. A. P. Hirt R. P. Lucocq J. M. Embley T. M. (2002). A Mitochondrial Remnant in the Microsporidian Trachipleistophora Hominis. Nature 418 (6900), 865–869. doi: 10.1038/nature00949 12192407

[B98] Yachnis A. T. Berg J. Martinez-Salazar A. Bender B. S. Diaz L. Rojiani A. M.. (1996). Disseminated Microsporidiosis Especially Infecting the Brain, Heart, and Kidneys: Report of a Newly Recognized Pansporoblastic Species in Two Symptomatic AIDS Patients. Am. J. Clin. Pathol. 106 (4), 535–543. doi: 10.1093/ajcp/106.4.535 8853044

[B99] Zhang G. Sachse M. Prevost M. C. Luallen R. J. Troemel E. R. Félix M. A. (2016). A Large Collection of Novel Nematode-Infecting Microsporidia and Their Diverse Interactions With Caenorhabditis Elegans and Other Related Nematodes. PloS Pathog. 12 (12), e1006093. doi: 10.1371/journal.ppat.1006093 27942022 PMC5179134

